# Integrated domestic violence and reproductive health interventions in India: a systematic review

**DOI:** 10.1186/s12978-024-01830-0

**Published:** 2024-06-29

**Authors:** Bushra Sabri, Serena Sloka Mani, Venkata Preetam Sandeep Kaduluri

**Affiliations:** 1 Johns Hopkins University School of Nursing, 525 North Wolfe Street, Room N530L, Baltimore, MD 21205 USA; 2 Johns Hopkins University Bloomberg School of Public Health, Baltimore, MD USA

**Keywords:** Domestic violence, Intimate partner violence, Gender-based violence, India, Reproductive health, Family planning, Interventions, Systematic review

## Abstract

**Background:**

Domestic violence is a leading cause of poor health outcomes during pregnancy and the postpartum period. Therefore, there is a need for integrated domestic violence interventions in reproductive health care settings. India has one of the highest maternal and child mortality rates. This review aimed to identify characteristics of existing evidence-based integrated domestic violence and reproductive healthcare interventions in India to identify gaps and components of interventions that demonstrate effectiveness for addressing domestic violence.

**Methods:**

A systematic review of intervention studies was conducted using Preferred Reporting Items for Systematic Reviews and Meta-Analyses. Three research team members performed independent screening of title, abstracts and full-texts.

**Results:**

The search resulted in 633 articles, of which 13 articles met inclusion criteria for full text screening and analysis. Common components of integrated violence and reproductive health interventions that were effective in addressing domestic violence included**:** psychoeducation/education (*n* = 5), skill building (*n* = 5), counseling (*n* = 5), engaging stakeholders with use of trained lay peer facilitators (*n* = 3), and engaging male spouses (*n* = 3).

**Conclusions:**

Interventions in India for domestic violence that are integrated with reproductive health care remain few, and there are fewer with effective outcomes for domestic violence. Of those with effective outcomes, all of the interventions utilized psychoeducation/education, skill building, and counseling as part of the intervention.

**Supplementary Information:**

The online version contains supplementary material available at 10.1186/s12978-024-01830-0.

## Abstract (plain language summary)

Domestic violence is a leading cause of poor health outcomes during pregnancy and the time after pregnancy. Thus, there is a need for integrated domestic violence interventions in reproductive healthcare settings. India has one of the highest maternal and child death rates. This review aimed to identify features of existing evidence-based integrated domestic violence and reproductive healthcare interventions in India to identify gaps and components of interventions that demonstrate effectiveness for addressing the problem of domestic violence among women in reproductive healthcare settings. A systematic review of intervention studies was conducted. The search resulted in 633 articles, of which 13 articles met the criteria to be included in this review. Common components of effective integrated domestic violence and reproductive health interventions included: psychoeducation/education (*n* = 5), skill building (*n* = 5), counseling (*n* = 5), engaging stakeholders with use of trained lay peer facilitators (*n* = 3), and engaging male spouses (*n* = 3). The key takeaways are that interventions in India for domestic violence that are integrated with reproductive healthcare remain few, and there are fewer with effective outcomes for domestic violence. Psychoeducation/education, skill building, and counseling were commonly used strategies in interventions that were effective in addressing domestic violence.

**Supplementary Information:**

The online version contains supplementary material available at 10.1186/s12978-024-01830-0.

## Background

Domestic violence (DV) is a global human rights and public health concern that has been associated with maternal morbidity and mortality, globally and in India [1–6]. Globally, the prevalence of domestic and related violence against women has ranged from 26–31% [7–9]. The United Nations has highlighted violence against women and girls under one of its Sustainable Development Goals (SDGs) as “Target 5.2: Eliminate all forms of violence against all women and girls in the public and private spheres, including trafficking and sexual and other types of exploitation.” [10]. The statistics included here use various and overlapping terms to define acts of violence against women and girls. They largely focus on spousal and intimate partner violence (IPV), as violence perpetrated by a husband or male intimate partner is the focus of most studies globally due to its widespread prevalence [9]. The United Nations defines domestic abuse, inclusive of IPV and DV, as “a pattern of behavior in any relationship that is used to gain or maintain power and control over an intimate partner. Abuse is physical, sexual, emotional, economic or psychological actions or threats of actions that influence another person. This includes any behaviors that frighten, intimidate, terrorize, manipulate, hurt, humiliate, blame, injure, or wound someone” [11]. The definition of DV in the Indian cultural context describes DV as the actual or the threat of physical, sexual, economic and/or psychological harm perpetrated on the woman by both spouse and/or in-laws [12–14]. This includes behaviors such as spreading of false rumors, social restrictions, controlled access to healthcare, control of reproductive rights, confinement to the home [15], dowry-related harassment and murders as well as honor killings [12, 14, 16, 17]. In India, the reported prevalence of domestic violence in nationally representative and community-based surveys has ranged from 32% to 77.5% [18–21]. DV during pregnancy can result in fatal and non-fatal adverse health outcomes for the pregnant woman and her child due to the direct trauma of violence to the woman’s body as well as the physiological effects of stress from ongoing or prior DV on growth and development of the fetus [22, 23]. Fatality or maternal mortality refers to a woman’s death during pregnancy, at delivery, or within 42 days of pregnancy loss, from any cause related to or aggravated by pregnancy or its management but not from any incidental or accidental reasons [4, 24]. Fatal outcomes of DV for women during pregnancy or postpartum care include both homicides and suicides [4, 22]. Non-fatal outcomes for the mother, or morbidities, include reproductive health issues such as low birthweight, preterm labor, insufficient weight gain, loss of a child during infancy, miscarriage, and physical and mental health problems such as injuries, depression, and impact on the child [22, 25–27]. In studies on DV during pregnancy in India, the prevalence has ranged from 22.2% [28] to 49.5% [29]. Further, DV during pregnancy and postpartum period in India has been associated with high perinatal, neonatal, and infant mortality rates [30].

Domestic violence during pregnancy (DVDP) and related types of violence have been associated with multiple adverse health outcomes such as hypertension [31] and labor and delivery complications [32]. DVDP has been linked to low birth weight [32–34], induced and spontaneous abortion [35], miscarriages [3, 6, 12, 36], stillbirth [37, 38], under-5 mortality [39, 40], and preterm labor [34, 41]. DVDP has also been linked to poor utilization of healthcare services such as delayed entry into antenatal care/immunization, decreased rest, and decreased food intake [42]. Moreover, DVDP affects breastfeeding practices, such as avoidance of breastfeeding [43], early termination of exclusive breastfeeding [44], reduced breastfeeding initiation, and shortened breastfeeding duration [45]. The mental health consequences of DVDP are also significant. This includes postpartum depression and suicidal ideation [46]. In a meta-analysis by Golding [47], the prevalence of post-traumatic stress disorder (PTSD) among victims of IPV during pregnancy ranged from 31% to 84.4%.

Reproductive healthcare services can be ideal settings for identifying and intervening with DV survivors in India to prevent DV-related maternal mortality and morbidity problems. However, healthcare professionals in India providing reproductive healthcare do not routinely inquire about DV during healthcare screenings or have the expertise to response to disclosures of DV [48]. Thus, there is need for evidence-based DV screening and intervention strategies integrated into reproductive health care settings in India to prevent maternal mortalities (fatal) and morbidities (non-fatal adverse reproductive health outcomes) among women in abusive relationships. Moreover, there is need to identify evidence-based DV approaches that can be integrated in reproductive healthcare settings in India. Therefore, the purpose of this study was to examine characteristics of evidence-based integrated DV and reproductive healthcare interventions in India that were effective in addressing DV and identify components of effective integrated DV and reproductive healthcare interventions that led to improved outcomes for DV survivors.

## Materials and methods

This review followed the guidance set forth under Preferred Reporting Items for Systematic Reviews and Meta-Analyses (PRISMA) guidelines [49].

### Search strategy

A search strategy was performed in the following electronic databases: PubMed, Global Health, and Embase. The extensive list of search phrases is presented in Appendix A. After a systematic search of articles with four key concepts: (1) India, (2) the time surrounding pregnancy and childbirth, (3) gender-based violence (GBV), domestic violence, and intimate partner violence, (4) interventions for DV, the search for articles was broadened in 2022 by two additional reviewers. During this process, the search criteria were broadened regarding the concept of pregnancy or childbirth to also include “reproductive health” and “family planning”. This allowed for inclusion of interventions that targeted DV and included any forms of reproductive healthcare. Integrated interventions were thus defined as those interventions that included at least one reproductive health component, inclusive of family planning, while also aiming to reduce DV. In addition to the search in the electronic databases, the team engaged in the process of handsearching using reference lists of existing systematic reviews on DV and reports in Google Scholar, as well as the reference list of selected articles.

### Eligibility criteria

The eligibility criteria for inclusion in the review were: (1) Quantitative or mixed-methods studies evaluating integrated DV and family planning or general reproductive health interventions, including women in prenatal, postnatal, and/or postpartum care. This included screening, prevention, and response interventions; (2) Studies using quantitative or mixed methods randomized controlled trials, non-randomized controlled trials, quasi-experimental or pre-post evaluation designs; (3) Studies that included women of ages 15 and older; (4) Studies conducted in India; (5) Studies published in peer-reviewed journals in English language from 2011–2022.

The exclusion criteria for the review were: (1) Studies that did not conduct a quantitative or mixed methods evaluation of an integrated DV and family planning or general reproductive health interventions, (2) Stand-alone DV intervention studies that did not have a family planning or reproductive health component. (3) Studies that did not report findings of an evaluation trial using quantitative or mixed methods experimental, quasi-experimental or pre-post designs. (4) Qualitative studies, literature reviews and study protocols. (5) Studies that included participants under the age of 15; (4) Studies conducted outside India; (6) Studies not published in peer-reviewed journals and not published in English language; and (7) Studies published before 2011.

### Study selection and extraction

All references from the electronic databases were imported into Covidence® [50], a software program for managing systematic literature reviews. Three reviewers independently conducted initial screening of titles and abstracts for all references. After screening conflicts were resolved, the remaining references underwent full-text review by the same reviewers for final eligibility. A data extraction sheet in excel was used to extract the information from the studies included in the final synthesis. After the data was extracted, the articles were classified by socio-ecological level of the mentioned intervention. A community-level DV intervention was designated as involving the desire to bring public awareness to the problem of DV within the community. It involved education of members of the community, the training of providers, or care within the community setting [51]. An interpersonal-level DV intervention pertained specifically to family-level relationships, most notably the intimate partner with which they may be engaging in the intervention [51]. An individual-level DV intervention focused on addressing individual’s attitudes and behaviors that influenced perpetration and/or victimization related to DV [51]. Finally, multilevel DV interventions included two or more of the above levels (e.g., community, interpersonal, and/or individual) delivered either at the same time or over time [52].

## Results

### Selected studies and characteristics

**Fig. 1:**
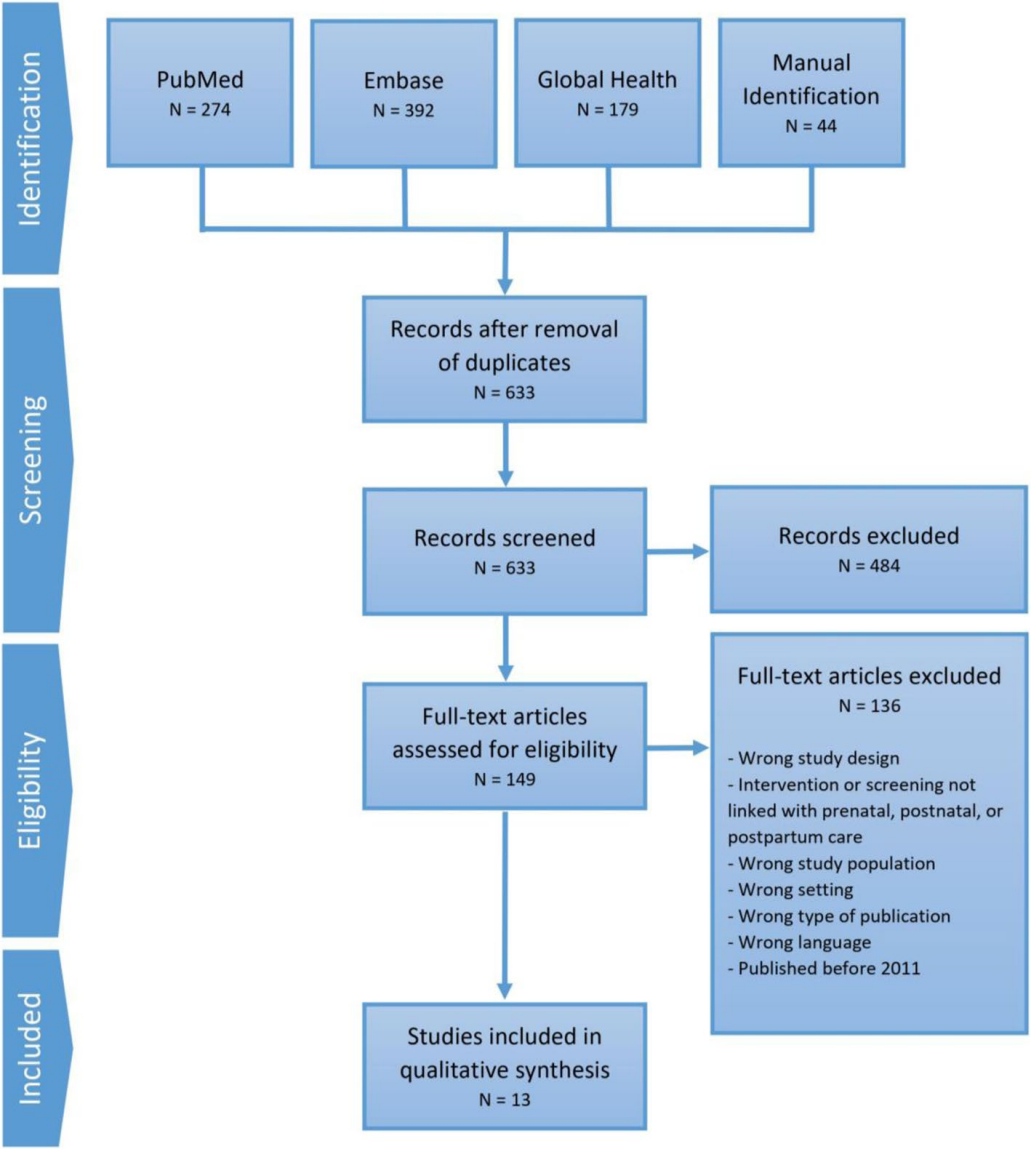
PRISMA flow diagram

The electronic search of the three databases identified 845 articles, with an additional 44 articles identified through hand searching. After removing duplicates (*n* = 256), the titles and abstracts of the remaining 633 articles were screened for inclusion and exclusion. The screening resulted in 149 possibly eligible full-text articles for which full text copies were retrieved and reviewed. The 149 full-text articles were reviewed based on the prespecified inclusion and exclusion criteria. This process resulted in the inclusion of 13 articles (11 interventions) for synthesis. The PRISMA flow diagram is presented in Fig. 1.

The summary of the selected characteristics of included studies is described in Table 1. The 11 interventions were evaluated using cluster randomized controlled trial (RCT; *n* = 5), pre-post (*n* = 4), quasi experimental (*n* = 1) or prospective non-randomized trial (*n* = 1) designs. The *ns* in the results present the number of interventions.

**Table 1:** Summary of studies included (*n* = 11 articles)

**Author**	**Intervention Name/Type**	**Theoretical Framework**	**State/Site**	**Design**	**Control Group**	**Sample**	**Violence Outcome**	**Follow up**	**Results for DV-related Outcome**
**Arora et al., 2019 **[53]	Counseling intervention in antenatal care settings/Empowerment based	Sullivan’s social and emotional well-being framework (Outcomes)	Maharashtra /Mumbai (urban)	Pretest-post-test	None	2515 women consented to screening with 408 reporting DV during pregnancy & participated in the intervention	Prevalence of DV during pregnancy; cognitive changes (understanding & perceptions of DV); change in women’s ability to cope, safety & health; Adapted Abuse Assessment Screen (AAS) for screening	6 weeks after delivery	Women reported cognitive changes in the range of 60–65% (e.g., recognizing the effects of violence and the need to speak out against it); There were no statistically significant changes in the steps taken to address DV. 36% of the women in the study used at least one safety precaution; At 6 weeks after delivery, 62% had recalled a safety measure; Significant decrease in financial, emotional, and physical abuse (statistically significance was not evaluated)
**Fleming et al., 2018; Raj et al., 2016 **[54, 55]	CHARM (Counseling Husbands to Achieve Reproductive Health & Marital Equity)	Social Cognitive Theory & Theory of Gender & Power	Maharashtra/Thane (rural)	cRCT	Standard government services	1081 couples from 50 geographic clusters (*n* = 467 couples; 25 clusters in CHARM & 614 couples; 25 clusters in control) (18–30 years of age & their wives)	Gender ideology (Gender Equity Men Scale); Equitable attitudes towards household decision-making [54]; IPV perpetration & acceptability questions from Demographic & Health Surveys [55]	Baseline, 9 & 18 months	In Raj et al. [55]; attitudes toward acceptability of DV significantly reduced over time among men & women were significantly less likely to report sexual IPV at follow up than the control arm.
**Javalkar et al., 2019 **[56]	Samvedana Plus/Reduce violence among sex workers’ relationships	Theory of change	Bagalkote district/Karnataka (rural)	cRCT	Waitlist	400 sex workers 18+ age with a current/recent intimate partner (last 6 months) from 47 villages (24 in Samvedana Plus & 23 in control)	Proportion of sex workers reporting any or severe physical or sexual IPV in the past 6 months: questions adapted from WHO Multicountry Study on DV & Women’s Health; IPV acceptance; disclosure of IPV to family & friends; improved knowledge of self-protection	Baseline (2014) Midline (2016) & Endline (2017)	No statistically significant reduction in physical/sexual IPV or severe physical/sexual IPV; Statistically significant reduction in acceptance of IPV & increased awareness of self-protection strategies (identifying allies & counter measures) among participants in Samvedana Plus
**Jones et al., 2013 **[57]	Reducing sexual risk among high-risk couples	Theory of reasoned action	Chandigarh (urban)	Pretest-post-test	None	30 couples (18–59 years); sexually active within 1 week; married; monogamous heterosexual relationship for 1 month or more; not pregnant & HIV/STI negative but at risk	Modified version of the Conflicts Tactic Scale (18 item)	Baseline/1 month post-test	Participants reported a statistically significant decrease in verbal aggression by their partners at post-intervention
**Kalokhe et al., 2021 **[58]	GBE (Ghya Bharari Ekatra)/ Couples intervention for primary prevention of IPV	Couples Interdependence Theory	Maharashtra/Pune (urban slum communities)	Prospective nonrandom design	Standard care	40 couples (20 GBE & 20 control) (18+ years of age; married for ≤ 1 year; cohabiting in a slum, chawl, or slum redevelopment community)	Abridged version of the Indian Family Violence & Control Scale (IPV (Past 1 month); Physical, sexual, psychological)	Baseline & 3 months	Fewer incidents of psychological abuse in GBE participants (3/17, 18%) versus control participants (4/16, 25%) at 3-month follow-up.
**Krishnan et al., 2016 **[59]	Namagaagi Naave/ Workplace intervention on attitudes & practices related to gender equity	Social Cognitive Theory	Karnataka/Bangalore (Urban)	Quasi-experimental	delayed control	1100 & 900 employees surveyed at 2 sites	Gender Equitable Scale for Women; Acceptability of physical violence against spouses; Awareness of support services	Baseline & 12 months after project initiation	Statistically significant improvements in attitudes related to unacceptability of IPV/more gender-equitable attitudes, more knowledge of IPV, Program implemented in partnership with a private garment company [No data gathered on IPV victimization & perpetration]
**More et al., 2017 **[60]	SNEHA (Society for Nutrition, Education & Health Action) Centre Model/Community Resource Centers	Adapted RE-AIM Framework	Maharashtra/Mumbai/(urban informal settlements)	cRCT	No intervention to the control group	Women of reproductive age (15–49) & children < = 5 years; 8271 women & 5371 children in SNEHA & 7965 women & 5180 children in control; 40 clusters each of around 600 households (12,614 households in the intervention & 12,239 in the control arm)	Number of consultations conducted for violence against women & children/No standardized measure reported	Before & after 2 years of implementation	Promising (fewer participants in the intervention arm (7%) reported past year IPV than those in the control arm (14%); violence-related services reported 314 consultations among intervention clusters; - Among women aged 15–49, less than 1% of those in the control group were aware of counselling services for survivors of violence against women and girls, vs. 24% in the intervention group. - Among women with children under age 5, none of the 3,800 in the control group were aware of counselling services for survivors of violence against women and girls, vs. 69 (2%) from the intervention group (not statistically significant)
**Nair, Donta, Shahina, 2020 **[61]	Intervention to reduce partner violence & enhance contraceptive use among women	Social Cognitive Theory & Theory of Gender & Power	Maharashtran/Mumbai (Urban Slums)	Pre-post test	None	1136 currently married women (aged 18–39 years), at least one child & unmet need for family planning recruited from 2 slums	Past 12 months IPV (physical, emotional & sexual violence) items from National Family Health Survey based on Modified Conflict Tactics Scale (CTS)	Baseline & 12 months	Overall, 52% reduction in IPV post-intervention; Among women who had not reported or women who had reported physical, emotional and sexual violence in the baseline survey 62.6%, 52.1% and 72.1% reported of no violence respectively in the post intervention survey. The difference was found to be statistically significant
**Nair, Daruwalla et al., 2020 **[62]	Community mobilisation through Participatory Learning & Action (PLA) coupled with access to prevention of violence against women and girls	Behavioral change theory	Jharkhand (Rural)	Pre-post test	None	Women registered with the 39 women’s groups of PLA meetings facilitated by ASHAs & active in the 22 intervention villages (679 women interviewed at baseline & 861 at endline)	Violence from the NFHS-4 women’s survey with additional questions added related to community violence (witch-hunting, social boycott & community violence)	Baseline & 16 months	83% of women reported that violence was unacceptable at endline, compared to 74.3% at baseline (adjusted *p* < .001) Proportion of women who experienced emotional violence from husbands in the past 12 months decreased (adjusted *p* < .001); Emotional & physical violence from family members decreased in the past 12 months (adjusted *p* < .001); Proportion of women seeking help for violence from family members increased
**Raj et al., 2013; Saggurti et al., 2014 **[63, 64]	RHANI Wives HIV intervention	Social Cognitive Theory, Theory of Gender and Power, Behavioral change theory	Maharashtra/Mumbai (Urban slums)	cRCT	Referrals to local social services for DV	220 women (18–40 years) living with a husband with alcohol problem & with lifetime physical & sexual IPV experience (118 intervention & 102 control)	Self-reported IPV (physical/sexual) in the last 90 days/3 months; Marital conflict & sexual coercion (No standardized measure reported)	Baseline & 4–5 month follow up	In Saggurti et al. [64]: Both the RHANI participants and the control group reported a significant decrease in marital conflict and marital IPV. But only RHANI participants have reported a decrease in marital sexual coercion
**Raj et al., 2022 **[65]	CHARM-2	Social Cognitive Theory, Theory of Gender and Power	Maharashtra (rural)	cRCT	Family planning brochure etc.	600 married couples; 20 clusters) Married couples residing together for at least 6 months; wife aged 18–29 years & neither spouse infertile/sterilized	Not reported	Baseline, 9 & 18 months	Violence outcome not assessed

*RCT* Randomized controlled trial, *cRCT* Cluster randomized controlled trial

### Setting

Most integrated DV-reproductive health interventions (7/11) were implemented and evaluated in the Indian state of Maharashtra in rural *(n* = 2; 53, 54, 55), slums (*n* = 3; 56, 57, 58, 59), informal settlements (*n* = 1; 60) and hospital settings *(n* = 1; 61). The slums and informal settlements were low-income settings, both urban and semi-urban areas characterized by poverty and substandard living conditions, majorly from Mumbai and Pune, the first and second largest cities in the state of Maharashtra. Two interventions were implemented and evaluated in Karnataka, one in an urban factory *(n* = 1; 62) and another in a rural area *(n* = 1; 63). Of the remaining interventions, one intervention focused on a rural area of Jharkhand [62] and another intervention [57] was implemented and evaluated in a hospital in the urban city of Haryana.

### Study population

For studies collecting data from individual participants, the sample sizes ranged from 220 to 1,136. For couples, the sample sizes ranged from 30 to 1,081. One study [60] collected data from 26,787 women and children from 12,614 households in the intervention arm and 12,239 in the control arm.

### Study groups

In four intervention evaluations using a pre-post design, there were no control groups [53, 57, 61, 62]. Two studies used a waitlist or a delayed control group who received the intervention at the end of the study [56, 59]. In five two-armed RCTs, the interventions were compared with the control groups which did not receive the intervention but instead received some supportive information of available services or referral to services [54, 55, 58, 63, 64] or received standard care available through public health care in the area [60, 65]. Of note, one of the above interventions also included street plays on the topic of partner violence for their control group [63, 64].

### Outcome measures

The violence-related outcomes assessed were gender equitable attitudes, acceptability of violence against spouses, and victimization or perpetration of physical, sexual, or emotional DV and other forms of violence. Two studies examined gender equitable attitudes using the Gender-Equitable Men (GEM) scale [54] or its adapted version for women (the Gender Equity Scale for Women) [59]. Acceptability of violence against spouses or attitudes towards DV were measured in four studies [55, 56, 59, 62] with one study reporting that the items on acceptability of DV were derived from Demographic & Health Surveys [55]. One study measured if the female participants recognized that violence was an issue of power and the impact of violence on health [53]. DV was measured using the modified Abuse Assessment Screen (AAS) [53] or Conflict Tactic Scale (CTS) items in the Demographic Health or National Family Health Surveys [55, 61, 62] or modified version of the CTS [57]. Two studies either adapted items from the World Health Organization (WHO) Multi-country Study on DV [56] or used a modified version of the Indian Family Violence and Control Scale [58].

### Measurement time points and length of follow-up

The duration of follow-up assessments for intervention evaluation studies ranged from 1 month to 3 years. The shorter follow up periods included studies that evaluated outcomes ranging from 1 [57], 3 [58] to 5 months follow up [63, 64]. For instance, in a study focusing on pregnant women, participants were assessed at first antenatal appointment and outcomes were evaluated at 6 weeks post-delivery [53]. The longer follow-up periods for evaluation ranged from 1 to 3 years. While some studies’ follow up periods ranged from 12 to 18 months [59, 61, 62, 65], others had a longer follow up period ranging from 2 to 3 years [56, 60].

### Intervention characteristics

Among the studies, five intervention studies reported a statistically significant reduction in at least one type of DV over time [55, 57, 61, 62, 64], with three studies reporting positive results from promising interventions [53, 58, 60]. Four studies reported statistically significant reduction in acceptability of DV [55, 56, 59, 62] or improvement in gender equitable attitudes in the household [54, 59]. The recruitment and/or implementation settings for these interventions were hospitals, antenatal care settings, infectious disease clinics, family planning clinics, community-based venues, or participants’ homes. Most of the interventions with statistically significant outcomes for DV were multilevel interventions [55, 61, 64], followed by interventions implemented at the interpersonal [57, 58], and community [62] levels. Almost all multilevel interventions engaged married couples [55, 61], except one [64] that was delivered to only women individually and in groups.

Specifically, the interventions that showed significant positive outcomes for future DV were *Counseling men to Achieve Reproductive Health and Marital equality* (*CHARM*) [55], *Raising HIV Awareness in Non-HIV-Infected Indian Wives (RHANI)* wives’ intervention [64], a sexual risk reduction intervention for couples [57], an individual and couples-based intervention for IPV and contraceptive use [61] and a community mobilization intervention [62]. The three promising interventions were *Ghya Bharari Ekatra* (“*Take a Flight Together*”) [58], the *Society for Nutrition, Education and Health Action* (SNEHA) [60] intervention and an *empowerment-based counseling intervention* in antenatal care settings [53]. Table 2 presents characteristics of interventions and the components.

**Table 2:** Characteristics of the interventions included

**Author**	**Intervention Name/Type**	**Components**	**Intervention Duration & Facilitators**	**Level of Intervention**	**Recruitment/Implementation Setting**
**Arora et al., 2019 **[53]	Counseling intervention in antenatal care settings	The intervention included several components, such as screening, information support, safety plan, skills-building, emotional support, supportive counseling, and access to community resources and social support. The information support involved providing the survivors with information about their rights and legal options. The skills-building involved teaching the survivors coping and problem-solving. The emotional support involved providing with empathy, validation, and encouragement.	Minimum 2 *individual* sessions, with additional sessions based on need (30–45 min each)- facilitated by trained counsellors	Individual	Public Hospitals/Gynecology Department/Antenatal Care Setting
**Fleming et al., 2018; Raj et al., 2016 **[54, 55]	CHARM//Gender Equity and Family Planning Intervention	The intervention included training, counseling, and services on family planning and gender equity. It also involved goal setting and action planning that assessed the level of marital violence and sexual communication among the couples and provided strategies to reinforce non-use of violence and promote respectful communication and interactions. The intervention also addressed gender equity-related issues, such as son preference, and encouraged healthy and shared family planning decision making.	[Total 4 sessions] 3 *individual* (married men) (Sessions 2–3 optional) & one couples’ *group* session -facilitated by male village healthcare providers) completed within 3 months	Multilevel	Clinical setting, or if required, near or in the participant’s home
**Javalkar et al., 2019 **[56]	Samvedana Plus	*Focus of groups with sex-workers (12 sessions)*: Reducing relationship risks; IPV education; Taking action against IPV; Changing norms; Safety planning & Skills building; *Individual counseling* with sex workers (as needed); *Group sessions with partners* (*8 sessions for 3 days):* IPV education, changing norms etc *Couple sessions* for those continuing to face relationship challenges. *Community:* village plays, monthly meetings to discuss cases of IPV	*Group* sessions/ trainings with sex workers facilitated by trained outreach workers; 8 participatory reflection modules implemented over 12 sessions.	Multilevel	Community
**Jones et al., 2013 **[57]	Reducing Sexual Risk Behavior among High-Risk Couples	*Group intervention* Cognitive-behavioral skills training; Role plays used participants’ experiences in problem-solving & cognitive restructuring- positive communication skills, conflict resolution & sexual negotiation—sessions covered gender-relevant issues (e.g., influence of in-laws, relationships); stress reduction/relaxation techniques (meditation/deep breathing): group sessions included practice, feedback, reinforcement; homework assignments	Gender-specific *groups* of 10 men or 10 women led by a gender-congruent counselors & peer facilitators-gender [3 weekly intervention sessions with each group (2 h/session] over 1 month	Interpersonal	Infectious Disease & Family planning Clinics
**Kalokhe et al., 2021 **[58]	GBE (Ghya Bharari Ekatra)/ Couples intervention for primary prevention of IPV	GBE plus a list of IPV & mental health support services to the female dyadic member: *Focus of Sessions* Communication/ conflict management, DV (subjective norms of IPV; discussion of the different forms & effects of IPV; exercise in which each participant ranked examples of violence by severity-factors individuals use to define acts of violence, highlighting individual-level differences in conceptualization of violence, & to challenge participants to expand their definitions of IPV and commit to a life of nonviolence. *Format*: Participatory intervention (Reflections, discussions, role plays, games, films, competition)	[6 session/ 6-week Group Intervention] Weekly 2-h *group sessions* over a 6-week period (6 sessions) with 3–5 newly married couples; 5 sessions facilitated by a male–female pair of trained peer educators with 6th session co-led by medical officers & delivered in gender-concordant groups [Two IPV/ DV relevant sessions]	Interpersonal	Community-based venue (e.g., school, community hall, Anganwadi, CBO)
**Krishnan et al., 2016 **[59]	Namagaagi Naave/Workplace intervention on attitudes & practices related to gender equity	The intervention aimed to address the intersections between gender norms, IPV, alcohol use, and reproductive and sexual health. It included a 1-week static standee exhibition near the dining area and cartoon storyline posters in the toilets that explained the campaign issue. The intervention also distributed informational flyers to each employee as they left for home on the second and third day, so that they could share the contents with their family members. On the fifth or sixth day, the intervention organized a 1-day mass awareness program in collaboration with referral service providers, using interactive street plays, experience-sharing sessions, interaction with experts, health camps, etc. It also created and strengthened referral linkages including training for NN Core team members as Margadarshis or Guides.	Week long	Community	Community/Factory
**More et al., 2017 **[60]	SNEHA (Society for Nutrition, Education & Health Action)	Center integrated counseling services to support survivors of physical, emotional, sexual, or economic violence by intimate or non-intimate partners. Women reporting violence are offered participation in an extensive support & response program that included crisis intervention with counselling, psychotherapy, and family intervention, and support with police complaints and legal redress	Home visits, Group meetings, community events facilitated by Community Organizers over 2 years organized around a SNEHA center	Multilevel	Community
**Nair, Donta, Shahina, 2020 **[61]	Intervention to reduce partner violence & enhance contraceptive use among women	[Total 4 sessions] 2 *individual* sessions with women followed by 1 *couple* session at home & an individual session with women. Family planning, marital communication, anger management, reducing risk related to violence, creating safe environment, and improving negotiation skills. The first two sessions were individual sessions (provided to women only), followed by a couple session (both husband and wife) followed by an individual session (provided to women only).	The sessions last for 2 months and has a gap of at least 2 weeks between each session. The sessions are about 30–45 min and are delivered by trained social workers to the women at their homes. The women are given action steps before each session.	Multilevel	Urban Slum communities
**Nair, Daruwalla et al., 2020 **[62]	Community mobilisation through Participatory Learning & Action (PLA)/ prevention violence against women and girls (SNEHA & Ekjut partnered intervention)	The two organization Ekjut & SNEHA partnered to implement a community-based intervention which included mapping of local services for the survivors, including women’s cell for counselling & First information Reports (FIR); Conducting sensitization workshops with law enforcement officers to help them understand their role in supporting the survivors; training ASHA’s to facilitate PLA. The PLA has four phases: Phase1 involved 6 group meeting where ASHAs used picture cards to educate on DV, workplace harassment, discrimination, witch hunting and role the of patriarchy; Phase 2 involved 3 group meetings where the groups analyzed causes of forms of violence and the consequences of DV through storytelling and games: Phase 3 involved % meetings focusing specific forms of violence prevalent in Jharkhand such as witch hunting and the available resources; Phase 4 involved 1 meeting about the achievements and continuation of the plan.	The intervention consists of 20–30 women in each group facilitated by ASHAs, who are accredited social health activists. These sessions are provided over a period of 16 months. The training provided by ASHAs is focused on domestic violence by a) fostering a sense of unity among survivors through Mahila mandals (women’s groups), Village Health Nutrition & Sanitation Committee meetings, & Gram Panchayat (village council) meetings; (b) helping in instances of violence by being alert to signs, providing shelter and emotional support & connecting women with health & legal services	Community	Village community
**Raj et al., 2013; Saggurti et al., 2014 **[63, 64]	RHANI Wives Intervention	The intervention included six sessions that covered topics such as relationship dynamics, sexual communication, Domestic Violence x, poverty, financial stress, and substance use. It used group discussions, counseling, and activities to educate and build skills among the women. The first two sessions (1&2) were individual sessions where they discussed financial stresses, health, alcohol use, and IPV. The third session (3) was a group session where they received education on HIV and marital communication. The fourth session (4) was another individual session where they talked about alcohol use, IPV, sexual violence, HIV, women’s health, empowerment, and safety. The fifth session (5) was another group session where they received group support and education on linkage to local services for HIV/STI, IPV, and alcohol. The sixth session (6) was the final individual session where they discussed alcohol use, sexual violence, HIV, and use of local support after the program.	4 household-based *individual* sessions & 2 small *group*-based community sessions delivered over 6–9 weeks facilitated by female counselors; Discussion based through stories, games; Problem-solving	Multilevel	Household & the community
**Raj et al., 2022 **[65]	CHARM-2	CHARM2 is a counseling intervention which expanded on the original CHARM intervention. They built in 5 gender-specific counseling sessions (at the individual level, two sessions for female participants provided by female health providers and two for male provided by male health providers followed by one couples' session at the interpersonal level). CHARM2 offered a wide range of contraceptive options like IUDs and OCPs through gender-matched healthcare providers in these sessions. Their approach was based on a proven person-centered care model that positions women alongside providers and male partners empowering them in family planning decisions Individual Level sessions for females focused on reproductive coercion, women's choice, fertility goals and discreet contraceptive use as necessary while the males' individual sessions focused on male engagement and respectful communication with wives; Interpersonal Level sessions focused on contraceptive communication and joint decision making, etc. for couples.	Total 5 sessions - 4 *individual* sessions (2 for women and 2 for men facilitated by gender matched healthcare providers) & one *couples* session completed within 6 months	Multilevel	Confidential setting; Community Center, Clinical Setting

#### Multilevel interventions

The four multilevel interventions that appeared to be promising or showed statistically significant reduction in DV over time were (a) an *integrated intervention to reduce partner violence and enhance contraceptive use* in urban slum communities [61]; (b) *a gender equity and family planning* intervention/Counseling men to Achieve Reproductive Health and Marital equality (*CHARM*) [55], (c) *Raising HIV Awareness in Non-HIV-Infected Indian Wives (RHANI)* Wives Intervention [64]; and (d) *Society for Nutrition, Education and Health Action* (*SNEHA*) [60].

The *integrated intervention to reduce partner violence and enhance contraceptive use* [61] aimed to reduce IPV and increase contraceptive use amongst women of lower socioeconomic status in the slums of Mumbai. The intervention consisted of four sessions over 2 months, with the first two sessions being individual sessions for women, the third session involving couples – both husband and wife, and the fourth session being an individual session for women. The focus of the sessions included family planning, marital communication, anger management, reducing violence-related risks, creating a safe environment, and improving negotiation skills. The sessions included education on topics such as family planning, anger management, and gender transformative approaches grounded in Social Cognitive Theory and the Theory of Gender and Power. Skill building occurred via building capacity to address various challenges as they arose. Women were requested to brainstorm how they would handle unhappy moments and to jot down strategies to address these moments. The sessions were counseling-focused and the integration of health promotion components was heavily addressed through family planning skill building. The intervention evaluation demonstrated a statistically significant reduction in physical, emotional, and sexual IPV at post-intervention follow up [61].

The *RHANI Wives* intervention [64], implemented in slum areas of urban Maharashtra, was a multisession (4 household-based individual and 2 community-based group sessions) intervention designed to reduce Human Immunodeficiency Virus (HIV) and IPV risk and enhance reproductive health in rural women. The intervention sessions were conducted over a period of 6 to 9 weeks at the homes of the women and other community locations. Participants were educated on topics such as IPV, financial stress and other risk factors for HIV transmission. Gender transformative approaches were utilized by addressing the link between gender inequities and marital violence. Women were engaged in brainstorming factors that led to their husband’s problematic behaviors. Psychotherapy involved analysis of in-depth root causes of violence, and a skill-building approach trained women on how to protect themselves, per their expressed desires. Gender empowerment counseling and support were embedded into the design of the intervention. The intervention was based on health promotion as it aimed to prevent the spread of HIV amongst non-HIV-infected wives. Women were also connected with neighborhood resources and networks for DV, alcohol abuse, testing, treatment, and counseling for HIV/STIs. Master’s-level trained counselors facilitated the intervention and were trained and retrained accordingly as needed. The intervention showed a significant and positive reduction in marital sexual coercion, which was not demonstrated by the control group. Marital DV – physical and sexual – and marital conflict was reduced significantly in both intervention and control participants [64].

The *CHARM* intervention [55], was a gender equality and family planning counseling intervention for husbands in rural Maharashtra. The 4-session intervention involved three sessions of gender equity and family planning counseling delivered by trained male village health care providers (VHPs) to married men separately and one joint session with their wives. The sessions were implemented in clinical settings or, if required, near or in the participants’ homes and were to be completed within a 3-month period. The intervention aimed to improve contraceptive use by reviewing negative attitudes towards contraception and reduce incident pregnancy and IPV in married couples. The intervention showed significant effects on increasing contraceptive use and communication and reducing experiences of sexual IPV. Further, participation in CHARM significantly reduced acceptability of DV among both men and women [55].

The *SNEHA* intervention model [60] established community resource centers delivering integrated activities to improve women’s and their children’s health in urban informal settlements in Mumbai, Maharashtra. The centers provided integrated sexual-reproductive health, child health and nutrition, and DV counseling services and support programs for women in abusive relationships. The strategies included home visits, group meetings, day care for malnourished children, community educational events and other services. Men and teenagers are also included in conversations and awareness-raising activities about sexual and reproductive health issues as part of the intervention. The model connected people to services like family planning, prenatal care, immunization, and violence prevention by way of referrals or connections. The intervention model appeared to be promising with some improvement in SNEHA participants versus those assigned to the control arm. Fewer participants from SNEHA centers (7%) reported past year IPV than those in the control arm (14%). However, the findings were not statistically significant [60].

#### Community-level intervention

The community-level integrated intervention that was effective for reducing DV was the *Community mobilization intervention for prevention of violence against women and girls* [62]; The *community mobilization intervention* [62] evaluation aimed to examine the preliminary effects of community mobilization as a vehicle to prevent violence against women and girls in Jharkhand, located in eastern India. Accredited Social Health Activists (ASHAs) facilitated the intervention which consisted primarily of participatory learning and action (PLA) groups. ASHAs from 22 villages underwent a 9-day training program consisting of three phases with each phase lasting 3 days. Following the training, the ASHAs facilitated a four-phase PLA cycle with groups of 20–30 women in their respective villages over a period of 16 months. In Phase 1, there were six group meetings held to discuss different types of violence against women and girls. Phase 2 involved storytelling and games within the groups to address the issue of violence, while also encouraging participants to brainstorm strategies for addressing it. Moving on to Phase 3, the focus shifted specifically to Jharkhand and topics such as witch-hunting were addressed. After completing 14 sessions, a larger community gathering was organized, which involved health workers and other relevant stakeholders. Finally, Phase 4 consisted of a meeting to assess the achievements of the intervention. The intervention resulted in a significant reduction in the proportion of women who reported emotional violence from husbands and physical and emotional violence from family members in the past 12 months. A significant greater number of women reported that violence was unacceptable at endline. There was also a greater increase in women seeking help for violence from family members [62].

#### Interpersonal level interventions

The two interpersonal-level interventions with at least one positive IPV outcome (significant or non-significant) (i.e., psychological, and verbal abuse) were couples-based interventions [57, 58]. These interventions were evaluated among 30–40 married couples using a pretest–posttest design and a prospective non-random design. The follow-up period ranged from 1–3 months. The sessions of these interventions lasted for 1 month to 6 weeks, with either 3 weekly group sessions (2 h per session) for 1 month [57] or once a week 2-h sessions over a period of 6 weeks [58] with a mix of separate groups for male and female spouses or couples together. The sessions were facilitated by trained gender congruent counselors or lay peer educators with couples’ group sessions facilitated by a male-female pair of trained peer educators. The common IPV components were education on IPV-related issues, and skills training in positive communication, problem solving, and conflict resolution. The activities included reflections, discussions, role plays, feedback, reinforcement, games, films, and homework assignments.

The interpersonal-level intervention with a significant reduction in IPV was a group-based cognitive behavioral intervention with integrated IPV and sexual risk reduction components, implemented in clinics among couples at risk for HIV/STIs in North India [57]. The intervention addressed sexual barrier use, HIV/STIs transmission, and the development of cognitive behavioral skills with a focus on sexual negotiation and communication. Men and women participated in three gender-specific group sessions a week, lasting 2 h, over 1 month. The sessions involved coping, skills training, and gender transformative approaches where men were engaged in learning non-coercive sexual communication, despite existing gender norms surrounding this concept. The intervention assigned homework to the couples and prioritized participatory learning to reinforce concepts. Strategies included practice, role plays, feedback, and reinforcement. Psychotherapy and skill building involved teaching active coping strategies, problem solving, and positive reframing. Counseling involved communication skill building and conflict resolution. HIV-related topics were integrated into the intervention. Counselors and peer facilitators facilitated the intervention and were trained by psychologists from both India and the United States. This integrated group-based cognitive behavioral intervention resulted in a statistically significant decrease in verbal aggression by partners at post-intervention follow up [57]. The *Ghya Bharari Ekatra* (“*Take a Flight Together*”) [58] couples’ intervention evaluation was a pilot study in which there were fewer incidents of psychological abuse in the intervention arm versus control participants, but the findings were not statistically significant [58]. The intervention evaluation occurred among couples in community-based venues (e.g., schools, community-based organizations) [58].

#### Individual-level intervention

The only intervention that was implemented at the individual level was an *empowerment-based counseling intervention* [53] for women in antenatal care. The intervention involved multiple approaches for supporting DV survivors such as screening using the adapted Abuse Assessment Screen, information and emotional support, counseling, access to resources for support and planning. The authors reported considerable reduction in physical, financial, and emotional abuse but the statistical significance of the findings was not reported in the results [53].

### Components of effective interventions for DV

Common components of effective interventions for reducing DV were psychoeducation/education on how to prevent DV, counseling (broadly defined as coaching on various topics) for informational or emotional support, or crisis situations and skill-building in areas such as healthy marital communication, problem-solving, conflict resolution, sexual negotiation, and coping. The gender transformative components included topics that raised awareness of and challenged gender inequitable attitudes, norms, values, and behaviors. Some included education on DV-related topics such as understanding DV, consequences of DV, reducing risk related to violence, creating a safe environment, and support services in the community. A variety of participatory learning approaches (learning through activities) were used such as role plays and games/storytelling to build rapport and intimacy between couples and to facilitate communication, and to identify strategies to address violence, practice exercises for active problem-solving, group discussions, feedback, reinforcement, and homework assignments. The interventions engaged both men and women in individual and/or group sessions and were delivered by trained facilitators. This included stakeholders (such as healthcare workers) and/or trained lay peer facilitators. The healthcare workers/providers were trained in assessing women for safety and concerns, making appropriate referrals, and developing safety plans or helping women with safety strategies for violence prevention. Integrated health components included reproductive health and contraceptive use literacy. Reproductive health literacy provided participants information on maternal and child health benefits of birth spacing, delayed first childbirth, family planning, adolescent sexual and reproductive health, and sexual and reproductive rights. Contraceptive use literacy included reviewing negative attitudes toward contraception, condom skills exercises, awareness campaigns, education, and counseling on contraceptive options to achieve these goals including: education regarding efficacy, risks, non-contraceptive benefits, and side effects of different types of contraceptives. Some interventions included additional health topics such as alcohol misuse, and HIV.

### Neonatal health results

Of the interventions in this review, two had a focus on neonatal health outcomes. Both interventions had promising outcomes for DV. Arora et al., 2019 found that out of all the observed pregnancies in the study, 89% resulted in live births and there were 3 instances each of preterm and stillbirths. Notably, the three stillbirths were preceded by an instance of physical violence [53].

In the study by More et al. [60], after the SNEHA intervention, the neonates who were breastfed within one hour of delivery had levels of childhood wasting that were comparable to the control group. However, there was an improvement in childhood wasting at the cluster level in the intervention group, indicating some positive effects of the intervention. The SNEHA intervention group also had higher rates of full immunization in children aged 12–23 months when analyzed per protocol. Thus, the SNEHA intervention was promising in reducing DV as well as in improving child health outcomes [60].

## Discussion

### Intervention characteristics

This review examined research on integrated DV and reproductive health interventions that addressed any IPV, DV, or related outcomes among women in India. Only 11 interventions were identified in India that conducted quantitative evaluations of integrated DV and reproductive health care interventions. Five of the 11 interventions were tested using a randomized controlled trial (RCT) [54–56, 60, 63–65], with the remaining tested using a pre-post, quasi-experimental or non-random design. Although 8 intervention models appeared to be promising or effective in addressing DV [53, 55, 57, 58, 60–62, 64], not all of them were evaluated using an RCT. Two of these evaluations had a short follow-up period ranging from 4 to 6 weeks [53, 57]. Moreover, only two out of the five rigorously evaluated interventions (i.e., RCTs) reported statistically significant findings in reducing DV [55, 64]. Thus, there is minimal body of evidence on rigorously evaluated interventions that integrate DV with reproductive healthcare in India.

The integrated interventions differed in format, content, settings, number of sessions offered, and inclusion criteria. Most of the interventions that appeared to be promising or effective for DV were evaluated in urban or rural areas in Maharashtra [53, 55, 60, 61, 64], except for two that were tested in rural Jharkhand [62] and clinics in Chandigarh, an urban area in North India [57]. The two rigorously evaluated interventions [55, 64] that reported positive outcomes for DV were both multilevel interventions implemented in rural and urban Maharashtra. This shows a critical need for more rigorously evaluated evidence-based integrated DV and reproductive health interventions in other regions in India.

Overall, most of the integrated DV-reproductive health interventions that were found to be promising or effective interventions for DV were multilevel interventions [55, 60, 61, 64] except two that were implemented at the community [62] or individual level [53]. The two rigorously evaluated interventions [55, 64] addressed partner violence and were multilevel interventions implemented in clinical settings, near or in participants homes, and/or in the community. Our findings are in line with a prior review that identified that most effective interventions in low- and middle-income countries were multilevel interventions [66]. Thus, it is important to consider levels beyond the individual when designing interventions for DV survivors. This entails moving from just addressing the needs of survivors of DV to interventions that also transform their relationships and address discriminatory social norms and systems that contribute to gender inequality and violence against women and girls [13, 67].

Common components of integrated DV-reproductive health interventions that were found to be effective in addressing DV were (a) *Psychoeducation/education (n* = *5),* awareness to challenge gender inequitable norms and to promote awareness of the health impact of DV and how to be safe and prevent future DV; (b) *Skill-building (n* = *5):* in multiple areas (c) *Engaging male spouses (n* = *3)* in the intervention; (d) *Counseling (n* = *5)* for information, emotional support, and crisis situations; € *Engaging stakeholders* (e.g., healthcare providers, law enforcement) *(n* = *3)*; *and* (f) *Using trained lay peer facilitators (n* = *3)*. The education and training received through integrated health components (e.g., sexual negotiation and communication and sexual decision making, and alcohol misuse) may have also been effective in addressing IPV because of the overlap of these issues in intimate relationships. For example, alcohol abuse has been found to be a significant contributing factor for frequent and severe abuse by a partner [17]. Therefore, an intervention addressing alcohol misuse can also lead to a reduction of alcohol-related violence in relationships. The findings on effective components for DV are in line with prior research [66] where psychoeducation, communication skills building, gender-transformative approaches and participatory learning were the most frequently included components of interventions that addressed violence against women in low-and-middle income countries [66]. Effective interventions also engage both women and men, address multiple drivers of violence, involve gender sensitization and empowerment activities, promote positive interpersonal relationships, use participatory learning methods, involve critical reflection and communication skills, and promote empowerment [66, 68].

### Importance of integration with reproductive health

Given the negative impact of DV on women in pregnancy and postpartum care, there is need for additional rigorous studies on interventions that cater to the needs of DV-affected women in reproductive healthcare services in India. Evidence from Arora et al., 2019 and other studies conducted in Hong Kong, USA and South Africa supports integrating routine screening, safety planning and empowerment counselling for DV with antenatal care. It has benefits in improving safety and health outcomes for the pregnant women [69–73]. WHO [74] recommends all pregnant women to be screened for DVDP and those who disclose DV should be offered empowerment counselling and advocacy support. WHO also outlines certain “minimum requirements” [74] for a provider to begin the conversation about potential partner violence. These minimum requirements are as follows – 1) having standardized protocols in place, 2) for the provider to have received adequate training, 3) privacy for the encounter, 4) the promise of strict confidentiality, and 5) a way to refer the individuals reporting partner violence to further services [74]. If these minimum requirements are met, the doctor’s office could be a safe and appropriate place for women facing DVDP to address their needs without the fear of judgement or retribution. It is imperative to create safe spaces for women to address their needs without fear; training health care providers to recognize the signs of DV and provide appropriate care along with a strong referral system to available legal, social and psychological resources will allow the health system to address IPV and other forms of violence against women during perinatal care.

There is a need for policies that mandate universal screening for DV in healthcare settings at the national level in India; the central government of India lacks a national strategy to this regard [75]. There is also a need for policies in India that support integration of DV prevention and response services with reproductive healthcare services, especially in underserved parts of India. This could include providing gender equity education to male partners who accompany their female partners to a perinatal visit, while also providing the female partners with skill building and counseling at the same point of care visit. Most of the reviewed interventions were tested in Maharashtra. This is consistent with a prior review that showed that only a few key states such as Kerala and Maharashtra have resources dedicated to creating more services for survivors of DV [75]. This highlights the need for policies that dedicate resources to support survivors in other regions across the country. Overall, programs are needed that address DV at a systemic level, rather than placing the onus solely on survivors of DV to seek out services.

### Future areas of research

More research is needed on interventions addressing the role of in-laws in perpetrating violence against women. Violence instigated and encouraged by in-laws is a major problem in India; yet, most of the studies in this review focused on partner violence. Further, the studies did not look at how different subgroups of women responded to the interventions in terms of factors like age, education, socioeconomic status, and the type and severity of violence, which may have important implications for tailoring and targeting the interventions. There is also a need to explore mechanisms through which integrated interventions influenced outcomes for DV-affected women in reproductive healthcare. By not exploring the potential mechanisms through which the interventions influenced outcomes, understanding of how or why these interventions worked or not worked is limited. Further, the cost-effectiveness, sustainability and long-term effectiveness of some of the included interventions have not been evaluated limiting their applicability and scalability. Thus, there is need for cost-effectiveness studies and scalability evaluations of interventions to help guide policy and practice choices, ensure their viability, and extend their reach in other settings and populations.

## Conclusion

The review addresses an important research question on the effectiveness of integrated DV and RH interventions in India, which fills a gap in existing literature and contributes to the evidence base for policy and practice. The findings on common components of effective interventions provides useful insights for designing and implementing future interventions.

Some of the limitations of this review are the inclusion of only studies published in English, thus missing the literature published in local languages. Furthermore, the studies included were only those published in peer reviewed journals that reported outcomes of the intervention. This excludes interventions from non-peer reviewed literature that are being implemented but have not yet been evaluated. There was lack of clarity of components in some articles as well as which components resulted in positive outcomes of the intervention. The review did not assess the risk of bias (potential for publication bias, selection bias, confounding bias, measurement bias, recall bias or social desirability bias) of the included studies affecting the validity and reliability. Despite the limitations, this study adds to the literature on integrated DV and reproductive healthcare interventions by providing key overview of components of interventions implemented at different socioecological levels.

### Supplementary Information

Supplementary Material 1: Appendix A. Search Terms and Concepts. Search terms and concepts used for this systematic review in Embase, Global Health, and PubMed.

## Data Availability

No datasets were generated or analysed during the current study.

## Abbreviations

AAS: Abuse Assessment Screen
ASHA: Accredited Social Health Activist
CHARM: Counseling men to Achieve Reproductive Health and Marital equality
CTS: Conflict Tactic Scale
DV: Domestic Violence
DVDP: Domestic violence during pregnancy
GBV: Gender-based Violence
GEM: Gender-Equitable Men
HIV: Human Immunodeficiency Virus
IPV: Intimate Partner Violence
PLA: Participatory Learning and Action
PRISMA: Preferred Reporting Items for Systematic Reviews and Meta-Analyses
RCT: Randomized Control Trial
RHANI: Raising HIV Awareness in Non-HIV-Infected Indian Wives
SDGs: Sustainable Development Goals
SNEHA: Society for Nutrition, Education and Health Action
STI: Sexually Transmitted Infection
VHP: Village Health care Provider
WHO: World Health Organization
