# Supplementary material for: Integrated domestic violence and reproductive health interventions in India: a systematic review
Source: Reprod Health. 2024 Jun 29;21:94. doi: 10.1186/s12978-024-01830-0 (PMC11218333; doi:10.1186/s12978-024-01830-0)
Supplement: Supplementary file 1 — Supplementary Material 1: Appendix A. Search Terms and Concepts. Search terms and concepts used for this systematic review in Embase, Global Health, and PubMed. [file 12978_2024_1830_MOESM1_ESM.docx]

**APPENDIX A**

**Embase search (N= 392)**

***Concept #1***

('India'/exp OR 'Andhra Pradesh'/exp OR 'Assam'/exp OR 'Bihar'/exp OR 'Chhattisgarh'/exp OR 'Goa'/exp OR 'Gujarat'/exp OR 'Haryana'/exp OR 'Himachal Pradesh'/exp OR 'Jharkhand'/exp OR 'Karnataka'/exp OR 'Kerala'/exp OR 'Madhya Pradesh'/exp OR 'Maharashtra'/exp OR 'Manipur'/exp 'Meghalaya'/exp OR 'Mizoram' OR 'Nagaland'/exp OR 'Odisha'/exp OR 'Punjab (India)'/exp OR 'Rajasthan'/exp OR 'Sikkim'/exp OR 'Tamil Nadu'/exp OR 'Telangana'/exp OR 'Tripura'/exp OR 'Uttar Pradesh'/exp OR 'Uttarakhand'/exp OR 'West Bengal'/exp OR 'Andaman and Nicobar Islands'/exp OR 'Jammu and Kashmir'/exp OR 'Chandigarh'/exp OR 'Lakshadweep'/exp OR 'National Capital Territory of Delhi'/exp OR 'Puducherry'/exp OR ('India' OR 'Assam' OR 'Uttar Pradesh' OR 'Andhra Pradesh' OR 'Arunachal Pradesh' OR 'Bihar' OR 'Chhattisgarh' OR 'Goa' OR 'Gujarat' OR 'Haryana' OR 'Himachal Pradesh' OR 'Jharkhand' OR 'Karnataka' OR 'Kerala' OR 'Madhya Pradesh' OR 'Maharashtra' OR 'Manipur' OR 'Meghalaya' OR 'Mizoram' OR 'Nagaland' OR 'Odisha' OR 'Punjab' OR 'Rajasthan' OR 'Sikkim' OR 'Tamil Nadu' OR 'Telangana' OR 'Tripura' OR 'Uttarakhand' OR 'West Bengal' OR 'Andaman and Nicobar Islands' OR 'Jammu and Kashmir' OR 'Chandigarh' OR 'Lakshadweep' OR 'Delhi' OR 'Puducherry'):ab,ti,kw)

AND

***Concept #2***

('maternal welfare'/exp OR 'maternal health service'/exp OR 'postnatal care'/exp OR 'Perinatal Care'/exp OR 'pregnancy'/exp OR ('maternal health' OR 'maternal health service*' OR 'Postnatal Care' OR 'Perinatal Care' OR 'pregnancy' OR 'maternal health' OR 'maternal health service*' OR 'Postpartum Care' OR 'Postpartum Program*' OR 'Perinatal Care' OR 'pregnant*' OR 'postnatal care'):ab,ti,kw)

AND

***Concept #3***

('domestic violence'/exp OR 'partner violence'/exp OR ' gender based violence'/exp OR 'battered woman'/exp OR ('Domestic Violence' OR 'Spouse Abuse' OR 'Gender-Based Violence' OR 'Intimate Partner Violence' OR 'Battered Woman' OR 'battered women' OR 'violence' OR 'abuse*' OR 'assault' OR 'trauma' OR 'abused spouse*' OR 'battered women'):ab,ti,kw)

**Global Health search (N= 179)**

**Concept #1**

"India" OR "Assam" OR "Uttar Pradesh" OR "Andhra Pradesh" OR "Arunachal Pradesh" OR "Bihar" OR "Chhattisgarh" OR "Goa" OR "Gujarat" OR "Haryana" OR "Himachal Pradesh" OR "Jharkhand" OR "Karnataka" OR "Kerala" OR "Madhya Pradesh" OR "Maharashtra" OR "Manipur" OR "Meghalaya" OR "Mizoram" OR "Nagaland" OR "Odisha" OR "Punjab" OR "Rajasthan" OR "Sikkim" OR "Tamil Nadu" OR "Telangana" OR "Tripura" OR "Uttarakhand" OR "West Bengal" OR "Andaman and Nicobar Islands" OR "Jammu and Kashmir" OR "Chandigarh" OR "Lakshadweep" OR "Delhi" OR "Puducherry"

**Concept #2**

"Postnatal Care" OR "Perinatal Care" OR "pregnancy" OR "maternal health" OR "maternal health service*" OR "Postpartum Care" OR "Postpartum Program*" OR "pregnant"

**Concept #3 (revised)**

"Battered Women" OR "Domestic Violence" OR "Spouse Abuse" OR "Gender-Based Violence" OR "Intimate Partner Violence" OR "Battered Woman" OR "abuse*" OR "assault" OR "trauma" OR "violence" OR "battered women"

**PubMed search (N= 274)**

***Revised* Concept #1**

("India"[mesh] OR "India"[tw] OR "Assam"[tw] OR "Uttar Pradesh"[tw] OR "Andhra Pradesh"[tw] OR "Arunachal Pradesh"[tw] OR "Bihar"[tw] OR "Chhattisgarh"[tw] OR "Goa"[tw] OR "Gujarat"[tw] OR "Haryana"[tw] OR "Himachal Pradesh"[tw] OR "Jharkhand"[tw] OR "Karnataka"[tw] OR "Kerala"[tw] OR "Madhya Pradesh"[tw] OR "Maharashtra"[tw] OR "Manipur"[tw] OR "Meghalaya"[tw] OR "Mizoram"[tw] OR "Nagaland"[tw] OR "Odisha"[tw] OR "Punjab"[tw] OR "Rajasthan"[tw] OR "Sikkim"[tw] OR "Tamil Nadu"[tw]OR "Telangana"[tw] OR "Tripura"[tw] OR "Uttarakhand"[tw] OR "West Bengal"[tw] OR "Andaman and Nicobar Islands"[tw] OR "Jammu and Kashmir"[tw] OR "Chandigarh"[tw] OR "Lakshadweep"[tw] OR "Delhi"[tw] OR "Puducherry"[tw])

AND

**Concept #2**

("Maternal health"[mesh] OR "maternal health services"[mesh] OR "postnatal care”[mesh] OR "perinatal care"[mesh] OR "pregnancy"[mesh] OR "maternal health"[tw] OR "maternal health service*"[tw] OR "postpartum care"[tw] OR "postpartum program*"[tw] OR "perinatal care"[tw] OR "pregnancy"[tw] OR "postnatal care"[tw] OR “pregnant”[tw])

AND

***Revised* Concept #3**

("Domestic violence"[Mesh:NoExp] OR “spouse abuse”[mesh] OR "gender-based violence"[Mesh] OR "intimate partner violence"[Mesh] OR "battered women"[Mesh] OR "domestic violence"[tw] OR "spouse abuse" [tw] OR "gender-based violence"[tw] OR "intimate partner violence"[tw] OR "battered woman"[tw] OR “battered women”[tw] OR “violence”[tw] OR "abuse*"[tw] OR "assault"[tw] OR "trauma"[tw])

Broadened Concepts:

Concept #1:

1. India
2. Assam
3. Uttar Pradesh
4. Andhra Pradesh
5. Arunachal Pradesh
6. Bihar
7. Chhattisgarh
8. Goa
9. Gujarat
10. Haryana
11. Himachal Pradesh
12. Jharkhand
13. Karnataka
14. Kerala
15. Madhya Pradesh
16. Maharashtra
17. Manipur
18. Meghalaya
19. Mizoram
20. Nagaland
21. Odisha
22. Punjab
23. Rajasthan
24. Sikkim
25. Tamil Nadu
26. Telangana
27. Tripura
28. Uttarakhand
29. West Bengal
30. Andaman and Nicobar Islands
31. Jammu and Kashmir
32. Chandigarh
33. Lakshadweep
34. Delhi
35. Puducherry

Concept #2:

1. Maternal Health Services
2. Prenatal Care
3. Postnatal Care
4. Perinatal Care
5. Reproductive Health
6. maternal health
7. maternal healthcare
8. maternal mortality
9. maternal morbidity
10. pregnancy
11. antenatal care
12. postpartum care
13. postnatal care
14. perinatal care
15. Reproductive health
16. Family planning

Concept #3:

1. Intimate Partner Violence
2. Spouse Abuse
3. Domestic Violence
4. Battered Women
5. Gender-Based Violence
6. intimate partner violence
7. domestic violence
8. spousal abuse
9. battered woman
10. gender-based violence
11. relationship violence
12. coercive control
13. domestic conflict
14. domestic abuse
15. domestic aggression
16. abused spouse
17. intimate terrorism
18. Trauma

Concept #4:

1. Intimate partner violence interventions
2. Reproductive health component
3. IPV interventions
4. Domestic violence interventions
5. Sexual violence interventions
6. Partner violence interventions
7. Intimate partner violence prevention
8. IPV prevention
9. Domestic violence prevention
10. Sexual violence prevention
11. Partner violence prevention
12. Reproductive health interventions
13. Intimate partner violence and reproductive health
14. IPV and reproductive health
15. Domestic violence and reproductive health
16. Sexual violence and reproductive health
17. Partner violence and reproductive health
18. Intimate partner violence programs
19. IPV programs
20. Domestic violence programs
21. Sexual violence programs
22. Partner violence programs
